# Autumn movements of fin whales (*Balaenoptera physalus*) from Svalbard, Norway, revealed by satellite tracking

**DOI:** 10.1038/s41598-020-73996-z

**Published:** 2020-10-12

**Authors:** Christian Lydersen, Jade Vacquié-Garcia, Mads Peter Heide-Jørgensen, Nils Øien, Christophe Guinet, Kit M. Kovacs

**Affiliations:** 1 Norwegian Polar Institute, Fram Centre, 9296 Tromsö, Norway; 2 CNRS, UMR CNRS - La Rochelle University, 7372 Villiers-en-Bois, France; 3 Greenland Institute of Natural Resources, Strandgade 91, 1401 Copenhagen K, Denmark; 4 Institute of Marine Research, Nordnes, P.O. Box 1870, 5817 Bergen, Norway

**Keywords:** Ecology, Animal migration

## Abstract

Insight into animal movements is essential for understanding habitat use by individuals as well as population processes and species life-history strategies. In this study, we instrumented 25 fin whales with ARGOS satellite-transmitters in Svalbard, Norway, to study their movement patterns and behaviour (Area Restricted Search (ARS), transiting or unknown) during boreal autumn/early winter. Ten of the whales stayed in the tagging area (most northerly location: 81.68°N) for their entire tracking periods (max 45 days). The other 15 whales moved in a south-westerly direction; the longest track ended off the coast of northern Africa (> 5000 km from the tagging location) after 96 days. The whales engaged in ARS behaviour intermittently throughout their southward migrations. During transit phases the whales moved quickly; one individual maintained an average horizontal speed of 9.3 km/h (travelling 223 km per day) for a period of a week. This study documents that: (1) some fin whales might remain at high latitudes during winter; (2) the whales that do migrate probably feed along the way; (3) they can maintain high transiting speed for long periods and; (4) one breeding area for this species is likely located in deep, warm water some 100 km west of Morocco.

## Introduction

Fin whales (*Balaenoptera physalus*) have a cosmopolitan distribution and are found in all major oceans from tropical to polar regions^1^. They are more common at high latitudes during summer and at low latitudes (normally not lower than 20°) during winter^2^, although some fin whales remain at high latitudes during winter or at low latitudes during summer^2^. Fin whales currently number approximately 79,000 individuals in the North Atlantic^3^ and appear to have recovered from the commercial whaling that took place in this area. There are even suggestions that the number of fin whales in the North Atlantic may be higher than pre-whaling numbers due to changes in the environment that have resulted in increased abundances of krill^4^. Seven stocks of fin whales are currently recognized by the North Atlantic Marine Mammal Commission (NAMMCO) for the North Atlantic—Eastern Canada, West Greenland, three central stocks that appear to mix (East Greenland—West Iceland—and East Iceland/Faroe Islands), Norway and Spain^5^. Morphometric data and genetics suggest that at very least the Northwest Atlantic and the Northeast Atlantic fin whales are separate populations^6^.

Tracking studies of fin whales in the North Atlantic are few, but the earliest attempts took place in the 1970s, using radiotracking with VHF technology that required tagged whales to be followed manually^7,8^. The first satellite tracking study was conducted in Icelandic waters in 1994. This individual remained southwest of Iceland throughout its 45 days tracking period^9^. Two fin whales tagged in Greenland (2000, 2001) shifted between inshore and offshore waters west of Greenland, with the longest track covering 1000 km (77 days)^10^. Another study equipped 11 fin whales with satellite tags in areas east and southeast of the Faroes Islands in 2000 and 2001^11^. Five of the tags were deployed from a helicopter, while the others were deployed in the more traditional manner, from boats. Only two tags reported positions (none of those deployed from the helicopter) intermittently for periods of 48 and 166 days, respectively. The whale with the shortest track resided on the Faroese shelf for the duration of the tracking period, while the other spent three weeks on the shelf, then moved southwest to about 46°N where it remained for three weeks before moving northeast to an area northwest of Ireland.

The most comprehensive studies of movements and space use by fin whales in the North Atlantic are from studies of animals equipped with satellite transmitters around the Azores Archipelago^12–14^. A high degree of sinuosity in the tracks of 12 fin whales tagged in the years 2009–2012 suggested that the whales were foraging close to the islands and nearby seamounts^12^. When they moved north, they travelled at high speeds (7.7 km/h) in almost direct trajectories until they reached areas north of 56°N, where they resumed feeding in areas between Iceland and Greenland. Another study, involving these same 12 whales in addition to four more animals tagged in 2014, analyzed environmental drivers of these large-scale movement^14^ and found that the whales were strongly associated with both static and dynamic environmental variables that are closely linked with productivity. The fin whales preferred areas with cold water and high biomass of zooplankton or warmer waters, while avoiding areas with intermediate temperatures during their northward migration. In addition, the fin whales used both coastal and oceanic areas, with their distributions being strongly influenced by mesoscale eddy activities. Such eddy areas have strong upwelling/downwelling processes that enhance productivity and create predictable areas for baleen whale feeding^14^. In addition to the studies mentioned above, eight fin whales have been tracked in the Mediterranean Sea; seven remained in the Mediterranean throughout their tracking periods while one individual migrated through the Strait of Gibraltar and spent some time south and west of Portugal^15,16^.

No fin whale tracking studies have been conducted in the Northwest Atlantic. Knowledge about fin whales in the Svalbard Archipelago, Norway, is scarce. The species is commonly observed in the area from March to November with a peak in the number of observations from June to September^17^. They are most commonly found along the shelf break on the west coast of the archipelago but are also often observed north of Svalbard as far as 81.5°N, sometimes even occurring in areas with loose drift ice^17^. Stable isotope studies of fin whales in Svalbard confirm that they feed at a higher trophic position than blue whales in the area, indicating that fin whales consume fish in addition to krill in this area^18^. During a ship-based survey in summer 2014, fin whales were estimated to number 563 (95% CI 241–1313) along the west coast of Spitsbergen (Survey Block ES1^19^). Although there are no winter observations available for this region, which has 4 months of polar night, fin whales are detected on passive acoustic monitoring devices in the Fram Strait between Svalbard and Greenland (at about 79°N) during the winter^20,21^.

The purpose of the present study was to use data from satellite tracking of fin whales to explore movements and habitat use during the autumn (and into winter), to expand our knowledge of this species in the North Atlantic and to investigate the hypothesis that some fin whales reside year-round in the High Arctic.

## Results

### Model performance and descriptive statistics

**Figure 1:**
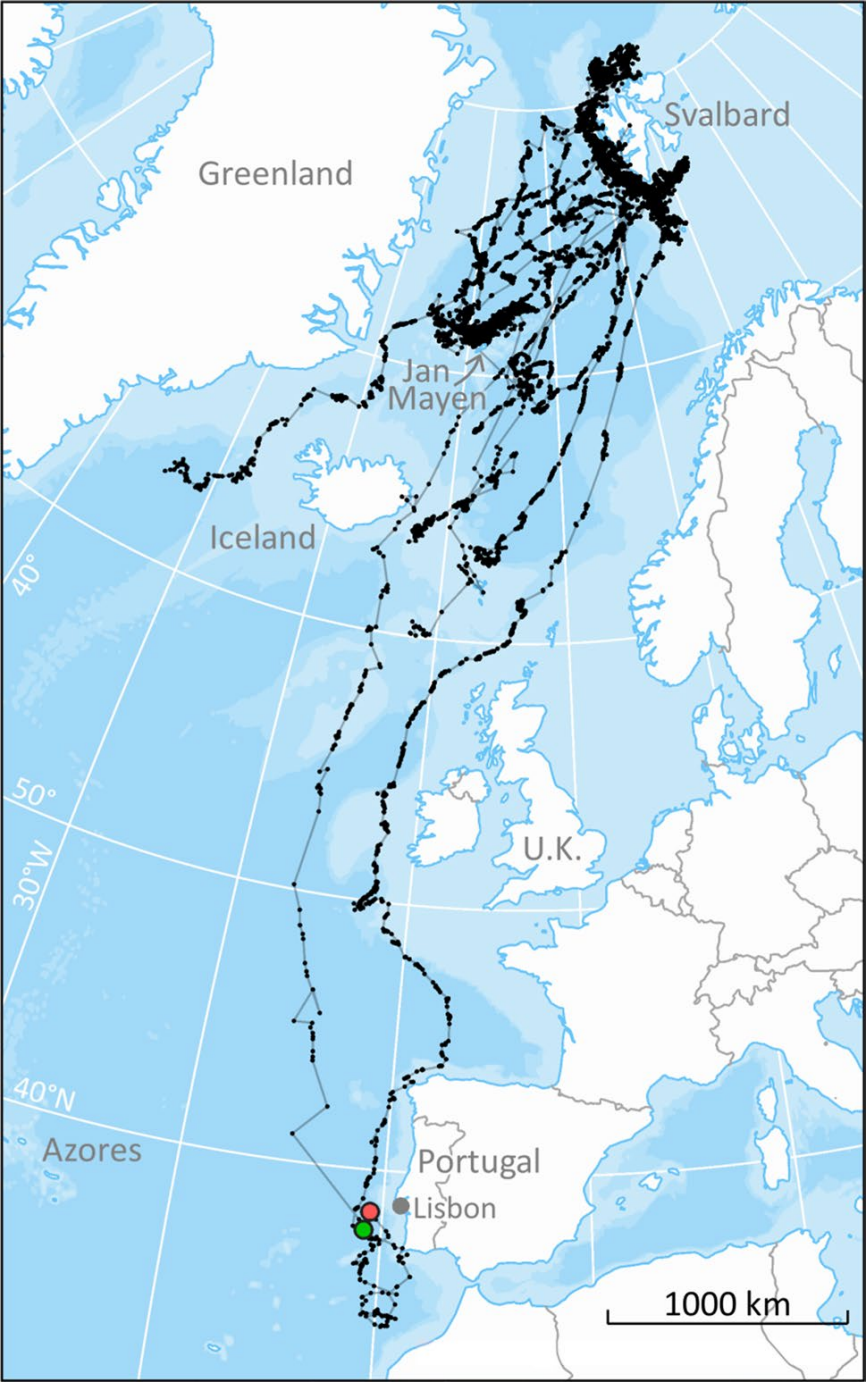
Argos locations, excluding Z location classes, received from 25 fin whales instrumented with satellite tags on the west coast of Svalbard during September 2015 (N = 2), 2018 (N = 4) and 2019 (N = 19). The green circle depicts the final location for one whale (ID 2015_1) received 21 November 2015. The red circle depicts the position for another animal (ID 2019_16) on the same date (21 November) in 2019. The map was generated based on publicly available ArcMap polar projections documents using ArcGIS 10.1 (www.esri.com).

The satellite transmitters deployed in this study on 25 fin whales in Svalbard provided a total of 14,469 locations, after the Z location classes were excluded, within tracking periods ranging between 6 and 96 d (average track duration 33 ± 4 days (± SE) see Table 1 and Fig. 1). The distribution of location classes was as follows: class 3—1%, class 2—2%, class 1–3%, class 0–4%, class A–20% and class B—70%. The average distance and duration between locations were 7.5 ± 0.1 km (range: 1 m–1186 km) and 1.4 ± 0.03 h (range: 1 s–6 days), respectively.

**Table 1:** Summary information for satellite tag deployments on 25 fin whales, instrumented in Svalbard, Norway, September 2015–2019.

Whale ID	Tag ID (PTT)	Deployment date	Date and time of first location	Date and time of departure from Svalbard	Track duration (days)	Number of ARS patches	Number of migratory segments
2015_1	93,100	9/18/2015	9/18/2015 15:55	10/11/2015 21:54	63.85	2	8
2015_2	93,102	9/18/2015	9/18/2015 14:39	ND	11.24	0	1
2018_1	24,640	9/10/2018	9/22/2018 16:08	ND	6.06	1	1
2018_2	20,685	9/10/2018	9/10/2018 21:45	9/20/2018 11:47	32.56	1	4
2018_3	93,097	9/10/2018	9/10/2018 23:58	9/10/2018 23:58	32.88	3	2
2018_4	24,641	9/10/2018	9/11/2018 16:54	9/12/2018 06:54	46.72	6	4
2019_1	21,800	9/21/2019	9/21/2019 12:12	9/22/2019 22:12	35.24	4	4
2019_2	21,802	9/21/2019	9/22/2019 17:01	ND	24.02	2	2
2019_3	21,803	9/21/2019	9/21/2019 09:01	ND	21.33	3	2
2019_4	22,849	9/21/2019	9/21/2019 16:53	9/23/2019 04:53	21.76	4	3
2019_5	37,278	9/21/2019	9/21/2019 08:48	ND	46.39	8	10
2019_6	93,106	9/21/2019	9/21/2019 12:21	ND	31.02	5	3
2019_7	168,435	9/21/2019	9/21/2019 11:54	9/24/2019 01:54	35.98	4	6
2019_8	168,449	9/21/2019	9/21/2019 16:55	9/23/2019 20:55	48.55	4	3
2019_9	168,455	9/21/2019	9/21/2019 11:44	9/29/2019 11:44	28.24	5	3
2019_10	168,458	9/21/2019	9/24/2019 06:11	9/26/2019 14:11	43.45	1	2
2019_11	168,460	9/21/2019	9/22/2019 07:48	ND	24.06	3	4
2019_12	168,463	9/21/2019	9/21/2019 15:12	9/24/2019 01:12	25.78	3	2
2019_13	168,464	9/21/2019	9/22/2019 08:20	9/23/2019 00:20	6.4	0	1
2019_14	168,465	9/21/2019	9/21/2019 08:31	ND	32.38	3	1
2019_15	168,466	9/21/2019	9/21/2019 12:03	9/26/2019 12:02	25.93	2	3
2019_16	168,469	9/21/2019	9/21/2019 11:44	9/23/2019 01:44	95.99	9	10
2019_17	168,470	9/21/2019	9/22/2019 07:48	ND	17.39	2	1
2019_18	168,471	9/21/2019	9/21/2019 16:50	ND	44.97	3	4
2019_19	168,472	9/21/2019	9/24/2019 05:59	9/24/2019 05:59	24.49	1	1

*ND* no departure, *ARS* area restricted search.

**Figure 2:**
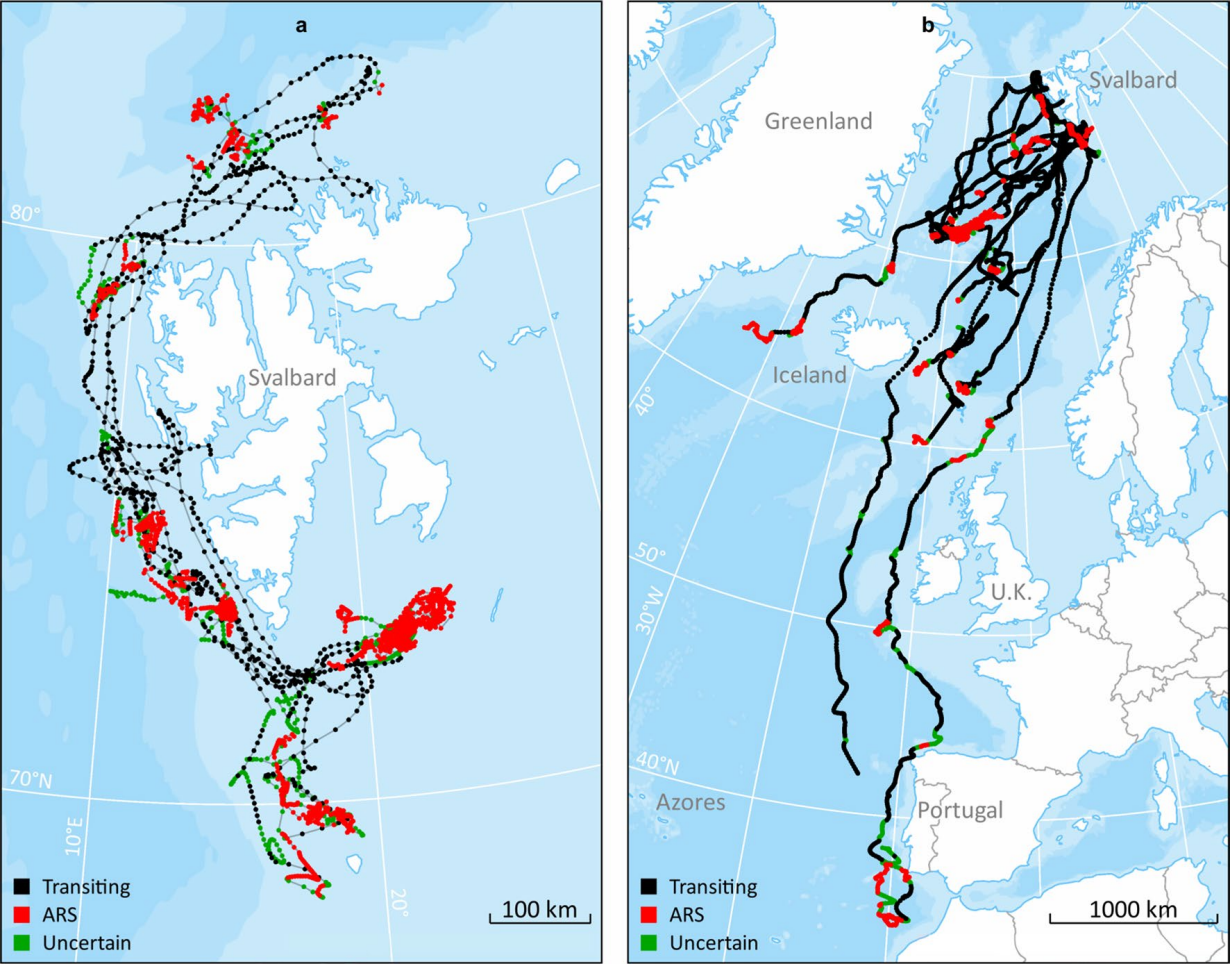
Hierarchical switching state-space model derived from raw Argos locations, and inferred behavioural modes, for 25 fin whales instrumented with satellite tags on the west coast of Svalbard during September 2015, 2018 and 2019. Panel a shows tracks of fin whales that did not leave the Svalbard area (N = 10), while panel b shows animals that underwent migrations beyond the Svalbard Archipelago (N = 15). The maps were generated based on publicly available ArcMap polar projections documents using ArcGIS 10.1 (www.esri.com).

Hierarchical Switching State-Space Models (hSSSM) detected two distinct behavioural states where the marginal posterior distributions of the movement parameter γ (speed and direction) were non-overlapping (Supplementary Fig. S1). The fin whales had higher γ during transit behaviour (median = 0.82; 95% CI: 0.80–0.84) compared to when they were engaged in Areas Restricted Search ((ARS) median = 0.009; 95% CI: 0.0004–0.04, see methods for more details regarding behavioural states). Extracting locations along the interpolated track segments at a 2 h resolution, produced 9662 locations from the hSSSM models. A total of 9640 locations from a total of 26 track segments were retained for analyses (22 locations that were estimated to be on land were removed (Fig. 2)). The fin whales performed ARS at 47 ± 5% of the locations (average per individual), with transiting occurring at 39% ± 5% of the locations; behaviour at 14 ± 2% of the locations was classified as being an uncertain state (Fig. 2). No ARS states were detected for two of the animals that had relatively short tracking periods (Individual 2015_2 and 2019_13, see Table 1).

### Whale tracks

Among the 25 fin whales equipped with satellite tags, 15 departed from the Svalbard area, while 10 were resident throughout their tracking periods. The last tag reporting locations from Svalbard did so on 6 November (Table 1, Fig. 2). Departures from Svalbard occurred between 10 September and 11 October (all whales tagged in 2019 left between 22–29 September). During the Svalbard residency phase for all the whales 49 ± 7% of locations per individual were in ARS, 22 ± 6% in transit and behaviour at 29 ± 6% of the locations was classified as being in an uncertain state. For the migratory phase (after animals left Svalbard), the corresponding numbers were 38 ± 6%, 50 ± 6% and 12 ± 2%, respectively.

**Figure 3:**
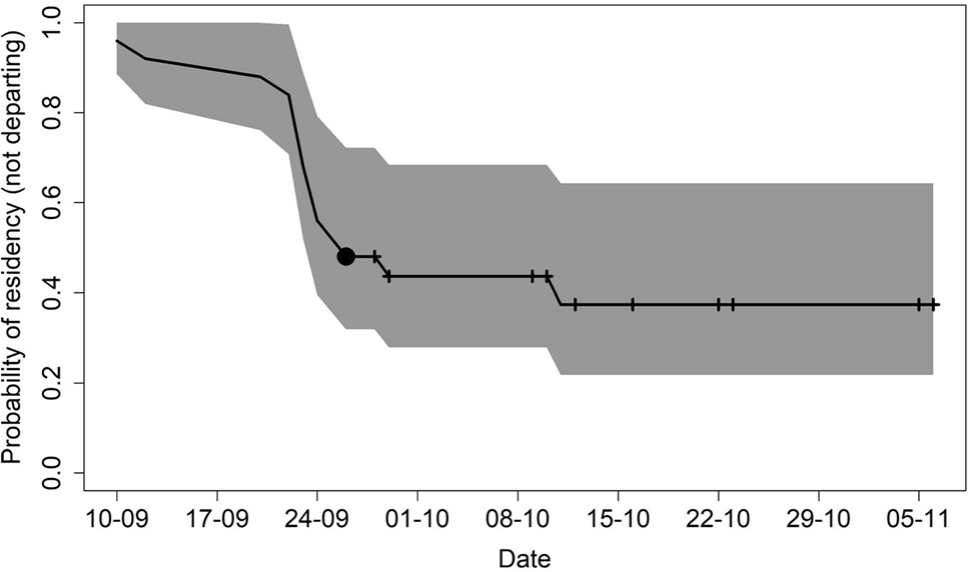
Probability of fin whales staying in Svalbard as autumn/winter progresses (residency time). The solid line represents the Kaplan–Meier estimate of the probability of fin whales not departing (1-probability of departing) from Svalbard, while the grey polygon represents the 25% and 75% estimated probabilities. Black crosses represent the observations for tags that stopped transmitting prior to a likely departure time. The median date for departure (the date at which 50% of the whales had departed) is indicated by the black dot.

The probability of staying in Svalbard decreased with day of the year (Fig. 3). The Kaplan–Meier survivor function estimated the median departure date to be 26 September; after 11 October the probability of leaving Svalbard was lower than 37% (Fig. 3). The whales that left the Svalbard area generally moved in a south-westerly direction, with the dispersion from their departure locations occurring in a non-random direction in September, October and November (Rayleigh test, 0.96, *p* value < 0.001, Rayleigh test, 0.86, *p* value < 0.001 and Rayleigh test, 0.96, *p* value = 0.01 for each of these months respectively; Supplementary Fig. S2). December was not tested since only one satellite tag was still transmitting positions, however this individual also headed in a southwestern direction (Supplementary Fig. S2). Several of the fin whales stopped travelling, and changed to ARS behaviour, when they were near Jan Mayen island (N = 3), and some tracks ended in this area (N = 2) (Figs. 1, 2). One fin whale swam through the strait between Iceland and Greenland, while the rest moved south between Iceland and U.K. (Fig. 1). The second longest track ended west of Lisbon (Portugal), while the longest track went as far south as Morocco. This latter whale subsequently moved north again, with its final locations being registered off the coast of Portugal (Fig. 1). These two tracks (4 years apart) overlapped off the southwest coast of Portugal in late November (Fig. 1). The longest track was approximately 5000 km in a straight line between the northernmost and southernmost positions. The whales that did not leave Svalbard reached latitudes north of 80°N (max 81.68°N; Fig. 2) in the late autumn.

During the time spent in Svalbard ARS behaviour was consistently high, occupying 60% of the whales’ time (Supplementary Figs. S3a, S4). In contrast, for migrating whales, the proportion of tracking time fin whales spent in ARS per day increased with the day of the year, with maximum values recorded in December (Supplementary Figs. S3b, S5). The whales spent more than half of their time in ARS in November and December, while in September and October they spent more time travelling (i.e. in transit); see the Supplementary Material for more details (Figs. S3b, S5).

**Figure 4:**
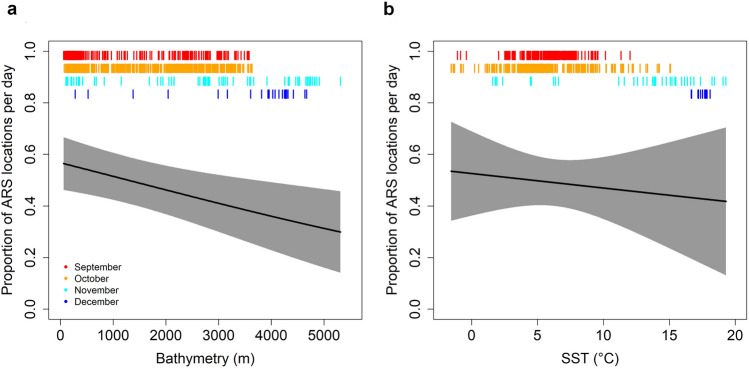
Effect of bathymetry (**a**) and Sea Surface Temperature (SST, **b**) on the proportion of estimated locations considered to be area-restricted search (ARS). Red, orange, light blue and dark blue bars at the top of each panel correspond to the distribution of Bathymetry and SST values for September, October, November and December locations, respectively.

Along their tracks, fin whales moved through areas characterized by bathymetry values from 2 to 5535 m (1499 ± 14 m), encountering SSTs ranging from − 1.8 to 19.3 °C (6.4 ± 0.04 °C) and distances to the coast ranging from 400 m to 655 km (146 ± 1 km). ARS occurred more often in shallower waters (< 2000 m) with low SSTs (< 10 °C) than in deeper waters with higher SSTs (Fig. 4), implying that bathymetry and SST are likely important variables for determining foraging areas. Temporal resolutions of 2 and 4 h were explored analytically, but both time frames produced similar results regarding the temporal variability in the ARS behaviour as well as habitat preferences, so only the 2 h intervals are presented herein.

**Figure 5:**
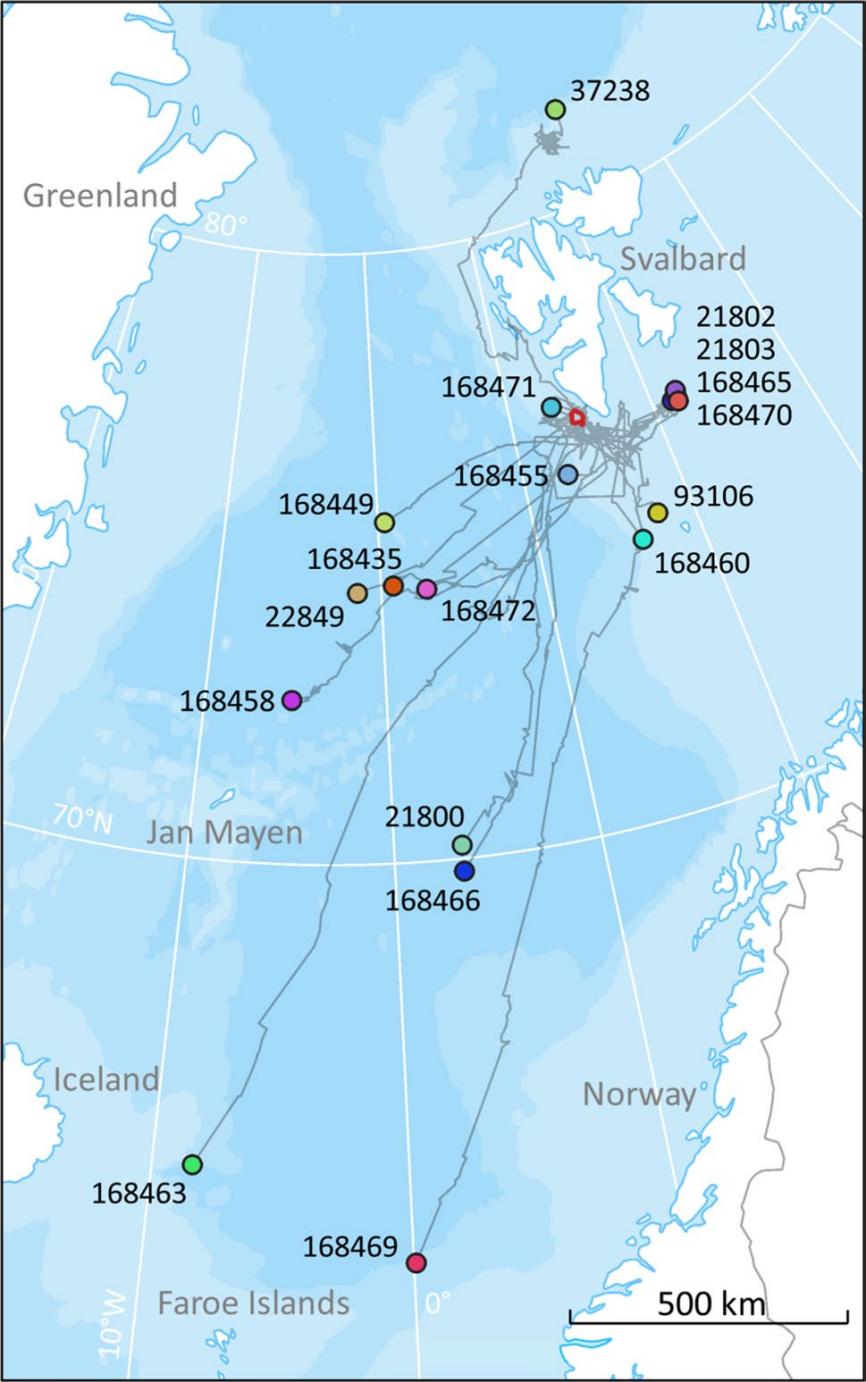
Map showing the tracks of 19 fin whales tagged with satellite transmitters within a 3 h period on 21 September 2019, within a small area southwest of Svalbard (red polygon). Circles of various colours represent these whales’ positions 10 days later (1st October 2019) and grey lines represent the paths followed. The map was generated based on publicly available ArcMap polar projections documents using ArcGIS 10.1 (www.esri.com).

Nineteen of the fin whales in this study were tagged from a helicopter within a restricted area (375 km^2^ polygon) in a period of less than 3 h on 21 September 2019 (Fig. 5). This provided an opportunity to explore group “coherence” in movement patterns. Ten days later (01 October) the group had dispersed in various directions over an area of more than 760,000 km^2^. The maximum distance between individuals in this 10 day time frame exceeded 2200 km (Fig. 5). One animal from the group moved up to 81.5°N (north of Svalbard), four individuals stayed relatively close to each other moving east of the tagging site, while most of the whales dispersed along individual paths (not travelling together) in a south-westerly direction reaching as far south as 63.2°N.

### Area restricted search patches and transiting phases

A total of 85 transiting phases were identified in the fin whale tracks (Table 1), with a mean of 3.40 ± 0.10 (± SE) per individual. These transiting phases lasted from 4 h to 17 days (mean 3.40 ± 0.40 days) and covered distances ranging from 8 to 2422 km (mean 415 ± 55 km). The speed during these phases ranged from 1.9 to 9.3 km/h (mean 4.7 ± 0.2 km/h). The maximum speed (9.3 km/h) was maintained for an entire week of travel by one individual, during which the whale moved 223.2 km per day.

**Figure 6:**
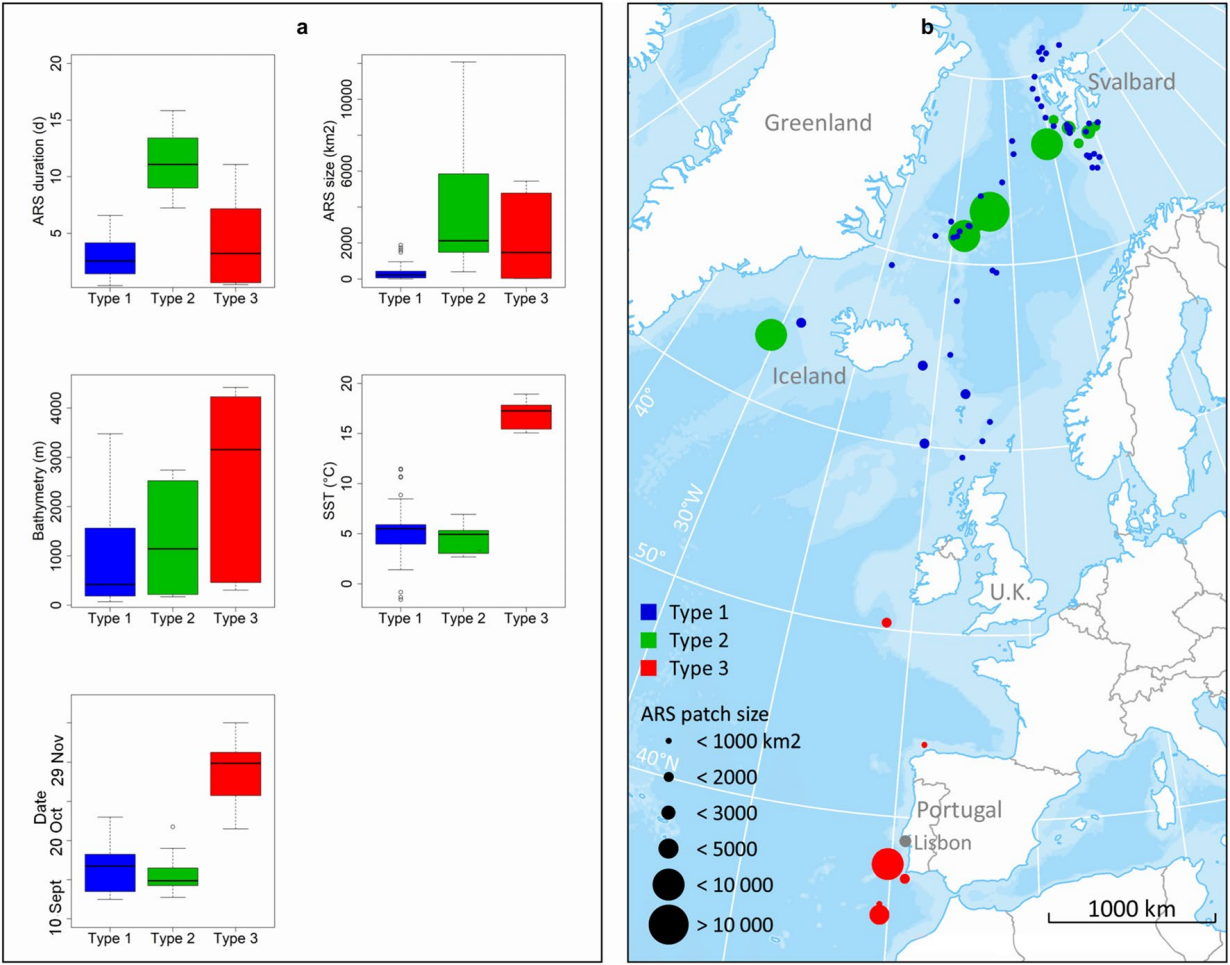
Boxplot showing duration, size, date, bathymetry (depth) and sea surface temperatures (SST) for three different Area Restricted Search (ARS) patch types that were identified via cluster analyses based on tracks from fin whales instrumented with satellite transmitters on the west coast of Svalbard (**a**). (**b)** shows the geographical distribution and size of the various ARS patches colour coded for type. The map was generated based on publicly available ArcMap polar projections documents using ArcGIS 10.1 (www.esri.com).

A total of 79 ARS patches were identified among 23 individuals corresponding to an average of 3.43 ± 0.09 (± SE) ARS patches per individual. Two whales showed no ARS behaviour—probably due to the short durations of their tracking periods (Table 1). Time spent in ARS mode lasted from 4 h to 38 days (mean 4.8 ± 0.62 days) and the size of the patches ranged from 1.8 to 12,079 km^2^ (mean 1053 ± 219 km^2^). The speed the whales travelled during ARS behaviour ranged from 0.27 to 2.40 km/h (1.36 ± 0.1 km/h). Exploring ARS behaviour using start date, DEP, SST, time spent in a given patch and size of the patch concomitantly, three different types of ARS patches were identified (Fig. 6). The first type (Type 1 hereafter; included 75% of the ARS patches) occurred mainly at the beginning of the tracks when the animals were close to the Svalbard Archipelago, Jan Mayen or between Iceland and the U.K. (Fig. 6a, b). Type 1 ARSs were characterised by short duration and small size (2.80 ± 0.22 days and 351 ± 57 km^2^, respectively), shallow bathymetry (1005 ± 136 m) and relatively cold SSTs (5.19 ± 0.35 °C) (Fig. 6a). The second type (Type 2 hereafter; included 18% of the ARS patches) also occurred mainly at the beginning of the tracks, in the same general area as the Type 1 patches (Fig. 6a, b). Type 2 ARSs were characterised by relatively cold SSTs (4.51 ± 0.35 °C) similar to the Type 1 patches (Fig. 6a). However, in contrast to Type 1 patches, Type 2 patches were characterized by longer durations, larger sizes (13.48 ± 2.10 days and 3518 ± 864 km^2^, respectively) and deeper bathymetry (1328 ± 302 m (± SE) (Fig. 6a). Finally, the third type (Type 3 hereafter; included 7% of the ARS patches) occurred mainly at the southernmost end of the tracks of the animals, in open ocean areas west of southern Portugal and Morocco (Fig. 6a, b). These ARSs had intermediate durations and sizes (mean 4.32 ± 1.69 days and 2204 ± 959 km^2^, respectively), and were characterized by deep bathymetry (2622 ± 774 m) and warm SSTs (16.96 ± 0.60 °C) (Fig. 6a). The warmest SST experienced in such an ARS was 19.3 °C.

**Figure 7:**
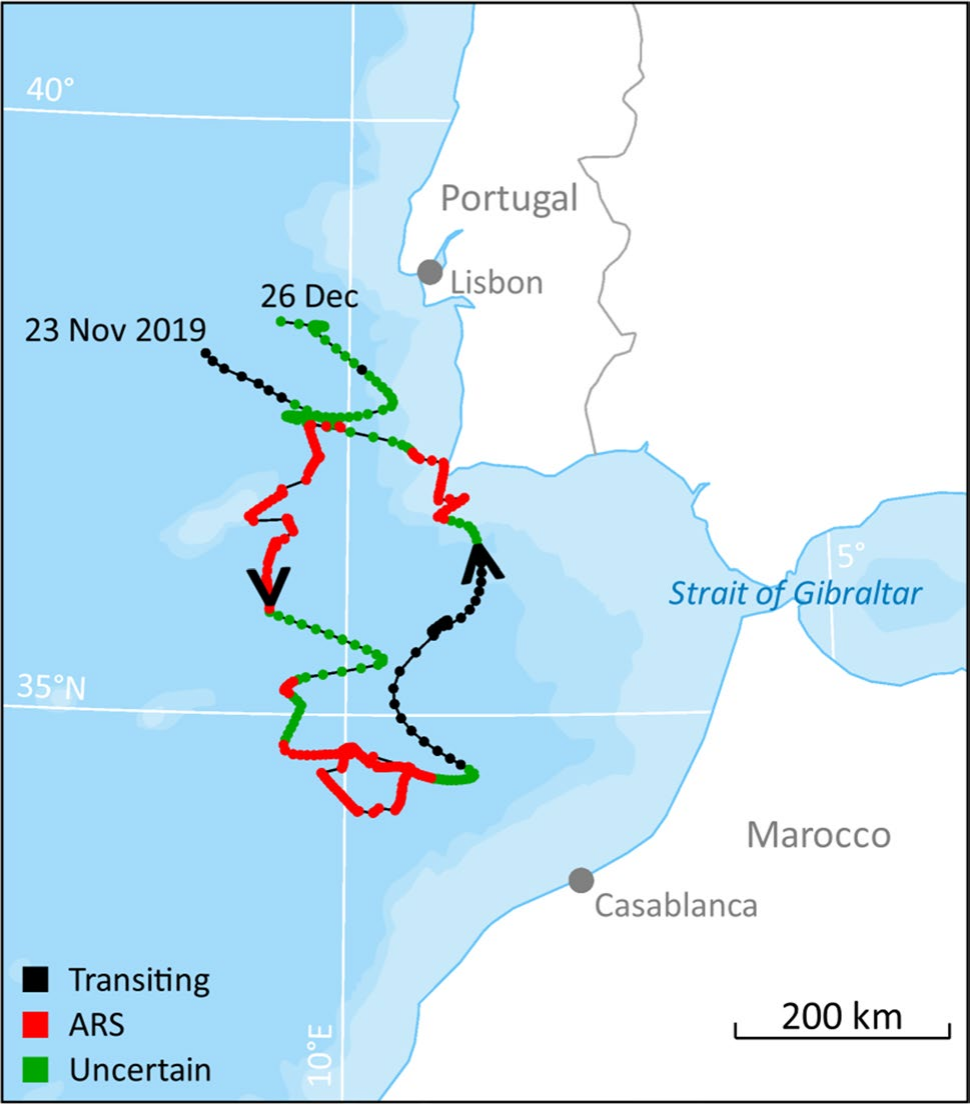
Hierarchical switching state-space model derived locations and inferred behavioural modes for the southernmost parts of a fin whale track (from 23 Nov to 26 Dec). This whale was equipped with a satellite transmitter off the coast of Svalbard, Norway, 21 September 2019. The map was generated based on publicly available ArcMap polar projections documents using ArcGIS 10.1 (www.esri.com).

One animal stopped its southward migration in an area about 200 km west-northwest of Casablanca in Morocco (Fig. 7). Here, it stayed for more than 11 days in a small ARS patch (Type 3) between 34 and 35°N. This area had an average depth of more than 4200 m and an average SST of 17.8 °C. On 17 December the whale started to move north again until the tag stopped transmitting on 26 December west of Lisbon, Portugal.

## Discussion

In this study we present novel information on movements and habitat use by fin whales in the North Atlantic and shed light on their migratory strategies and behaviour during the autumn and early winter. The whales were instrumented with satellite tags in Svalbard in September, and the majority initiated south-westerly migrations starting between 10 September and 11 October. Some of the whales did not depart during the time the tags transmitted (Fig. 2a). One possibility is of course that they departed later in the autumn or winter. However, since the tags stopped transmitting, their wintering locations cannot be verified. However, the latest location from a whale that was classified as not leaving Svalbard was 6 November, which is three weeks after the last whales that were known to migrate left Svalbard, after the onset of the Polar Night. Additionally, there is definitive evidence from passive acoustic recorders, one of which is close to 79 °N that detect fin whales in northerly areas throughout winter^20,21^. Similar information is also available for areas around Iceland, where fin whales are observed during winter^22^, and from Davis Strait where acoustic recordings have documented the presence of fin whales throughout the winter^23^. One of the fin whales that stayed in Svalbard was tracked up to 81.68°N, which represents the most northerly location documented for this species.

The animals that stayed in Svalbard transited up and down the west coast of Svalbard, and some also visited the north and south sides of the archipelago. Most of the time in the Svalbard area (57 ± 8% of all recorded positions) was spent in the ARS state (Fig. 2a). Even if ARS might represent a variety of behaviours, including resting^24^, it is reasonable to assume that these animals foraged much of the time ARS was recorded given observed behaviour of fin whale in the Svalbard area in summer and autumn. Additionally, many of the ARS patches documented in this study are located over shelf breaks close to known fishing banks in the area.

The average departure date from Svalbard in this study was 26 September, but this is likely heavily skewed by the fact that 19 of the 25 tags were deployed on a single day in 2019 and the migrating whales from this group all left within a week. It is likely that there is considerable interannual variation in when fin whales leave the Svalbard Archipelago given the spread in departure dates for the other years in this study. During migration 41% ± 5 of all recorded positions were spent in the ARS state (Fig. 2b). The whales generally swam in a southwesterly direction. One animal moved through the strait between Iceland and Greenland, but the main migration corridor was between Iceland and U.K. None of the migrants showed any transatlantic tendencies as had been expected, they remained in the Northeast Atlantic. The proportion of tracking time the whales spent in ARS per day was higher in shallower waters (< 2000 m) and areas with low SST (< 10 °C) than in deeper waters or in areas with higher SST. The association between ARS locations and marked bathymetric features known to characterize whale foraging habitats such as submarine canyons and continental shelf edge, i.e. slope structures and cold temperature that can induce upwelling and enhanced productivity, suggests that most of the documented ARS locations, associated with both the migratory phase and with residency around Svalbard, are likely foraging areas. ARS areas were particularly concentrated around Jan Mayen and Iceland. Thus, there is strong circumstantial evidence that the fin whales feed *en route* to their southern breeding grounds. Fin whales have also been found to feed prior to departing wintering grounds (e.g. around the Azores) before they initiate their northward migrations during spring^12,25^, so the general concept that these whales feed only when at their northern summer grounds, is likely incorrect.

The two longest tracks both went through the “corridor” between Iceland and U.K., but they differ in many other ways. The most westerly track (ID2015_1, Fig. 1, Table 1) belongs to a fin whale that left Svalbard 11 Oct 2015 and moved south to the east coast of Iceland. Thereafter it moved far offshore more or less straight south until it approached the coast of southern Portugal, where the tag stopped transmitting. This whale travelled quickly and ARS behaviour was not detected during its southward migration (Fig. 2b). The other track (ID2019_16, Fig. 1, Table 1) belonged to a fin whale that left Svalbard 21 September 2019. It travelled much further to the east, following the edge of the continental shelf north and west of the U.K., travelling closer to the European coast as it moved south to Portugal. This second whale stopped numerous times along its track, among other places at a site southwest of Ireland where blue and fin whales are known to congregate during autumn^26^. It is important to note that the frequency of the raw locations is different between these two tracks (Fig. 1) and that this could have created some bias regarding their comparison. ARS behaviours along the western track could have been hidden by the low resolution of the track, although if ARS behaviour had been common in that track, the frequency of raw locations would have been higher. Despite the low sample size, these two very different tracks suggest that the strategies for southward movements may vary between individuals and could be a consequence of factors such as body condition, sex, presence of food patches along the route and breeding status.

During the transit phases between ARS patches, the fin whales moved with speeds ranging from 1.9 to 9.3 km/h. Fin whales are fast swimmers, maybe the fastest of the baleen whales, and horizontal speeds based on tracking data have previously been reported between 5.38 and 11.11 km/h^7–9,27^. In addition, Aguilar and Garcia-Vernet^1^ suggest maximum speed for short bursts can be up to 15 knots (27.8 km/h) and Gambell^28^ suggests 20 knots (37.0 km/h). The maximum speed in the present study (9.3 km/h, 223.2 km per day) was sustained for an entire week, with the animal travelling more than 1500 km during this period. The real speed of the animal must have been considerably higher, given that our calculations are based on straight line 2-d movements, while in reality the whale swims in a 3-d environment with dives and surfacings along a curvilinear path. Long periods of high-speed travel are likely a factor that contributes to the relatively short track durations in the present study, since the drag on the external parts of the satellite transmitter must be high. The same tag design was recently used on the much slower swimming bowhead whale (*Balaena mysticetus*) (also deployed from helicopter) that had an average track duration of 181 days, with the longest duration being 709 days^29^.

The high travelling speed is also reflected in the dispersal of the individuals that were all tagged within a small area on the same day (Fig. 5). At this site, a large (but unknown) number of fin whales, occurring as single individuals or in small groups, were loosely spread over an area of a few kilometers. Except for one humpback whale (*Megaptera novaeanglia*), only fin whales were observed from the helicopter during this tagging event, and all tagged animals were large and thus likely adult individuals. Subsequent to tagging, the animals spread over an enormous area (760,000 km^2^). Four of the tagged whales moved more or less together to the southeast of Svalbard (Fig. 5), but the others apparently travelled alone. Fin whales are not considered to be gregarious and are normally found singly, in pairs or small groups, but they are reported to occur in temporary aggregations of a hundred or more in areas with high food abundance^1,28^. It was obviously such a feeding aggregation that we found at Svalbard. The ship’s echosounder confirmed dense patches of potential prey in the upper 100 m of the water column; acoustic experts confirmed that the prey patches consisted of small schooling fish, likely a young year-class of some gadoid species. Stable isotope studies of fin whales from this area suggest that fin whales feed on a mixed diet of krill and fish^18^.

The Type 3 ARS patches occurred almost exclusively at the southernmost reaches of the whales’ migrations, off Portugal and the coast of Morocco. They occurred in the period that is thought to be the breeding period for the species, and thus likely represent areas where breeding (mating and calving) occurs. The warm waters associated with Type 3 ARSs would be advantageous for the thermoregulation of newborn calves. However, whether warm water is crucial for calf survival is an issue open for debate. Some authors have suggested that the long migrations to lower latitudes by large baleen whales are related to predator avoidance (e.g.^30^). According to Aguilar and Garcia-Vernet^1^, no definite wintering grounds (and thus breeding areas) are known for fin whales in the North Atlantic. These authors suggested that due to the influence of the Gulf Stream (warmer water) suitable winter conditions are likely found over a large latitudinal area in the North Atlantic. Some of the fin whales in the present study certainly performed long latitudinal movements, with the longest over 5000 km in straight line from the northernmost to the southernmost location. A recently published study using 24 ocean-bottom seismometers over a large area (from about 35–37°N and 8.5–11.5°W) off the southwest coast of Portugal documented that fin whales are present in this area from November to January^31^. They suggest that this area is a breeding area for Atlantic fin whales. Similarly, based on acoustic detections, fin whales were shown to be present in areas around the Azores from the autumn through until spring^32^, and a logical assumption is that this is another area used by fin whale for breeding. In addition, we suggest based on the present tracking studies that the deep, warm open water area some 100 km west of Morocco likely is also used for breeding by fin whales.

Most of the fin whales in this study (23 out of 25) were tagged from a helicopter. This approach for deploying tags has been attempted before with limited success^14^. In the present study it was found to be a very efficient method.

In summary, the present study has shown that some fin whales that summer far north in the Atlantic Ocean might also stay in these northern areas during the winter. When in the north the whales alternated between ARS and transiting behaviour indicative of foraging and moving between different foraging patches. Some of the whales undertook long migrations in a south-westerly direction, the longest trip being more than 5000 km to the coast of northern Africa. Most of the whales engaged in ARS behaviour along their southward tracks, indicating that they stop and forage on their way to the breeding grounds. During the transit phases, the whales moved quickly; one individual maintained an average horizontal speed of 9.3 km/h for a period of a week. Based on the locations from one individual that started to move north again after a period of 11 days at its southernmost position, we suggest that the deep, warm, offshore waters some 100 km west of Morocco is likely a calving ground for fin whales.

## Methods

### Transmitter deployments and data collection

Fieldwork was conducted on the west coast of Svalbard during September 2015, 2018 and 2019. Fin whales (N = 25, Table 1) were instrumented with Wildlife Computers Spot 5 satellite transmitters (https://wildlifecomputers.com/) deployed using an ARTS (Air Rocket Transmitter System^33^) air gun (12–14 bar pressure) from a distance of 5–10 m. The Spot 5 transmitter included one AA-lithium battery together with the electronics in a stainless-steel casing (tube: 110 × 22 mm) with a stainless-steel stop plate (38 mm in diameter) 3 cm from the distal end. The stop plate prevents the tag from penetrating deeper into the blubber. The stainless-steel tube was attached to an anchor spear equipped with a sharp-pointed tip and foldable barbs along the spear. The total length of the tag from the stop plate to the tip of the anchor was 170 mm and the weight of the instrument, with attachment spear, was 133 g. The whales were either approached at sea using a small boat (Polarcirkel 845 with 2 × 150 hp outboard engines, N = 2) or from the air using a helicopter (Eurocopter 350 Ecureuil, N = 23). The SPOT 5 tags were programmed to make 250 transmissions per day starting at 0600 GMT. Given the relatively short time the transmitters were expected to be remain attached to these fast swimming whales and the fact that we were mainly interested in where the whales moved when they left the Svalbard area (if they did so), fieldwork was conducted as late in the year as weather was likely to allow for tagging operations, i.e. between 10 and 21 September (Table 1). On one occasion (21 Sept 2019) a large, “loose” aggregation of many fin whales was found southwest of Spitsbergen; 19 animals were instrumented from the helicopter during a period of 3 h (including a refueling of the aircraft and crew).

The Spot 5 tags collect and transmit information on location via the Argos satellite system (for details; see^34^). Location data were processed with the Kalman Filter by CLS Argos^35^. Z location quality classes were removed prior to further analyses due to the high uncertainty around them.

### Switching state-space model

A Bayesian switching state-space model (SSSM) was applied to the Argos-derived telemetry data to assess movement and behaviour of the fin whales^36,37^. The principle of a state space model is to estimate, at fixed time intervals, the state of an unobservable process from an observed data set. In the particular case of a SSSM, state refers to (1) the animal locations and (2) the behavioural states (in our case—Area Restricted Search (ARS) vs transiting^38,39^). A SSSM is composed of a process model that predicts the future state (location and behavioural state of an animal) given its current state, and an observation model that links the unobserved location states predicted by the process model to the observed data. In other words, the process model estimates movement parameters (including the combined autocorrelation ɣ) of speed and direction (i.e. movement persistence) that allow animals to move from one location to another, while the observation model corrects the tracks by taking into account the irregularity and variable errors in the observed Argos locations (see^36,37^ for full details). A first-difference correlated random walk model (DCRW) was used as the process model to describe movement dynamics and allow for movement parameters to switch between two discrete behavioural states (ARS vs transiting) by including a process model for each of them (a distinct DCRW for each behavioural state with its distinct values for the movement parameters). The switching process is governed by a Markov Chain Model that describes the behavioural states through time by estimating the probability of switching states from transiting at time *t* to ARS at time *t* + 1^37^. In the present study, the SSSM was fitted under a hierarchical framework (hSSSM) by assuming individuals are ‘samples’ from a population^40–42^. The advantage of this approach is that it combines information from all tracking data, resulting in a more efficient parameter estimation^43^. In addition, these models enable parameter estimation even for short/incomplete tracks, by analysing these together with data from other tracks.

The hSSSM was run using the R package *bsam* via JAGS 4.2 (Just Another Gibbs Sampler, created and maintained by M. Plummer (https://mcmc-jags.sourceforge.net)). Tracks with data gaps > 4 transmitting days were split into 2 or more segments since tracks with large gaps result in poor model fits^44^. Tracks or segments of tracks that were shorter than 3 transmitting days were removed. Priors for movement parameters were chosen based on previous studies^12,36,37,44,45^. Based on the resolution of the Argos derived location data, the hSSSM was fitted using a time step of 2 h (comprising 90% of time steps recorded and corresponding to the mean duration between locations). A lower temporal resolution (4 h) was also tested to ensure that the results were independent of scale. The hSSSM ran two parallel Markov Chain Monte Carlo simulations (MCMC), each comprising 180,000 iterations. The first 100,000 samples were discarded as a burn-in, and the 80,000 remaining samples were thinned by retaining every 40th sample to reduce autocorrelation effects. Thus, the model parameters and estimates of the whales’ locations and behaviours were calculated based on a total of 4,000 MCMC samples. Model convergence and sample autocorrelation were assessed by visually inspecting trace and autocorrelation plots, which showed good model convergence.

### Analysis of whale tracks

Whale locations and behaviour at 2-h and 4-h were inferred from the output of the hSSSM. In order to take into account all of the bimodal MCMC samples [1 (transiting) or 2 (ARS)] for the behavioural state, the final behavioural state was estimated at each location as the mean value of the MCMC samples. The same process was done for the longitude and latitude at each location. Then, using the same procedure as described in Jonsen et al.^46^ the following cut-off points were used: locations with mean estimates of behavioural state < 1.25 were assumed to represent transiting, locations with mean estimates of behavioural state > 1.75 were assumed to represent ARS and locations with mean estimates of behavioural state between these values were considered ‘‘uncertain’’.

Three environmental variables, bathymetry (DEP, m), sea surface temperature (SST, °C) and distance to the nearest coast (COAST, km), were calculated for each of the extracted locations. DEP was extracted from the 0.01-degree resolution ETOPO 1 Arc-Minute global relief data set from the National Geophysical Data Center, NOAA^47^. SST was extracted from the monthly 2° grid resolution from the Extended Reconstructed Sea Surface Temperature (ERSST) v5^48^, while COAST was calculated using the land file (1:10 m) from www.naturalearthdata.com. Locations estimated to occur on land were removed.

Departure times from Svalbard for the fin whales were identified following Lesage et al.^44^ as a consecutive period of ≥ 48 h of hSSSM-predicted transiting behaviour (i.e. mean estimates < 1.25). However, given the large size of the coastal areas around the Svalbard Archipelago, a condition relative to distance from Svalbard’s’ coastline was added to Lesage et al.’s definition^44^. Departure was therefore identified as the first period of ≥ 48 consecutive hours of hSSSM-predicted transiting that occurred further away than 150 km from the coastline. Individuals that stayed closer than 150 km from the coastline, even if they had more than 48 consecutive h of hSSSM-predicted transiting during this period, were not considered to have departed from Svalbard. The 150 km threshold was determined by a bimodality in the distribution of the maximum distance to the coast reached by the whales. The time fin whales spent in Svalbard after instrumentation (but before departure—hereafter called residence time) was then investigated in relation to the day of the year (i.e.: number of days after 10th of September which was the first deployment date in this dataset). To achieve this, a non-parametric Kaplan–Meier estimator of survival probability was fitted to these two temporal parameters following^13^. This analysis offers the advantage of including a departure probability for all individuals, including those for which tags stopped transmitting prior to being classified as having departed Svalbard. Monthly dispersions from the departure point (i.e. bearing from departure location to the last position in each month—*argosfilter* package in R) were then calculated for each individual that departed, separately. A Rayleigh test was used to test whether the directionality of their monthly dispersion was random.

Temporal variability in the ARS behaviour, as well as habitat preferences, of fin whales was then investigated using Generalized Additive Mixed Models [GAMMs (‘*gamm*’ function in the R package *mgcv*)]. Variation in the proportion of tracking time fin whales spent in ARS per day was examined in relation to (1) the day of the year and (2) the oceanographic parameters, separately. For assessing the temporal variability in the ARS behaviour (which might reflect feeding in one location/time frame and breeding in another), resident and migratory phases have been studied independently. Oceanographic parameters were initially averaged per day for each individual. Only DEP and SST were included in the analyses since COAST and DEP were highly correlated. A quasibinomial error distribution and a logit link function were used in the models and “individual” was included as a random effect and as a grouping factor in the first order autoregressive (corAR1) structure to minimize the effects of the hierarchical structure of the data. Model selection and model validation were done using the confidence intervals of the corresponding smoothing curves, as recommended by Zuur et al.^49^.

### Identification of the area restricted search patches and migratory phases

Migratory phases and ARS patches were identified for each individual. A migratory phase was defined to begin after 3 or more successive locations in which transiting behaviour was predicted (behavioural estimated state < 1.25) and to end after 3 or more consecutive locations with a behavioural estimated state ≥ 1.25. An ARS patch was defined to begin after 3 or more successive locations in which ARS behaviour was predicted (behavioural estimated state > 1.75) and end after 3 or more consecutive locations with a behavioural estimated state ≤ 1.75^24^.

Habitat preferences of the fin whales were explored by examining each identified ARS patch according to day of the year they began and the mean value of DEP and SST in which they occurred. Time spent in an ARS and the size of this patch (cf using minimum convex polygon—km^2^) were also calculated. To avoid collinearity problems between the variables, a principal component analysis (PCA) was conducted first and subsequently a hierarchical clustering analysis was applied on the five resulting principal components^50^. All of the ARS patches were thus classified hierarchically into groups according to the distances between them using the “Ward.D2” method (*hclust* function in the R package *stats*—^51^). Statistically different groups resulting from this analysis correspond to separate types of ARS patches.

### Animal ethics

Animal handling protocols were approved by the Norwegian Animal Research Authority (permits refs: 2015/28335-2, 17/20161, 19/16624) and the Governor of Svalbard (permits refs: 2015/00353-2, 16/01532-8, 16/01600-9) and carried out in accordance with the relevant guidelines and regulations.

## Supplementary information

Supplementary Figure 1.

Supplementary Figure 2.

Supplementary Figure 3.

Supplementary Figure 4.

Supplementary Figure 5.

Supplementary Legends.

## Data Availability

The datasets analyzed during the current study are available in the Norwegian Polar Data Centre repository at: https://data.npolar.no/dataset/d46e3394-d99c-4fb3-a298-1259edf15ca3.
