# Supplementary figures and images for: Autumn movements of fin whales (Balaenoptera physalus) from Svalbard, Norway, revealed by satellite tracking
Source: Sci Rep. 2020 Oct 12;10:16966. doi: 10.1038/s41598-020-73996-z (PMC7550606; doi:10.1038/s41598-020-73996-z)

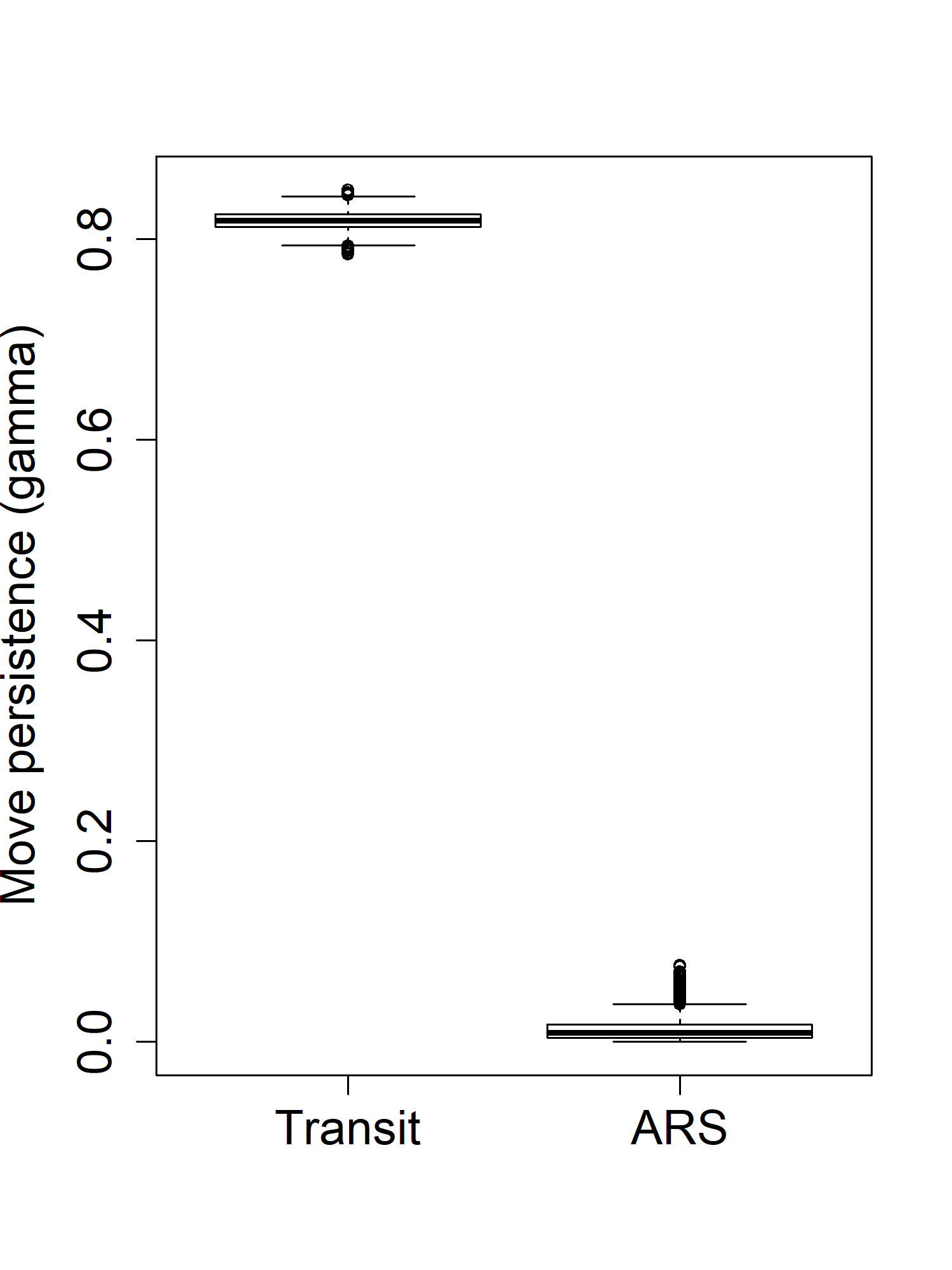

Supplement: Supplementary file 1 — Supplementary Figure 1. [file 41598_2020_73996_MOESM1_ESM.tiff]

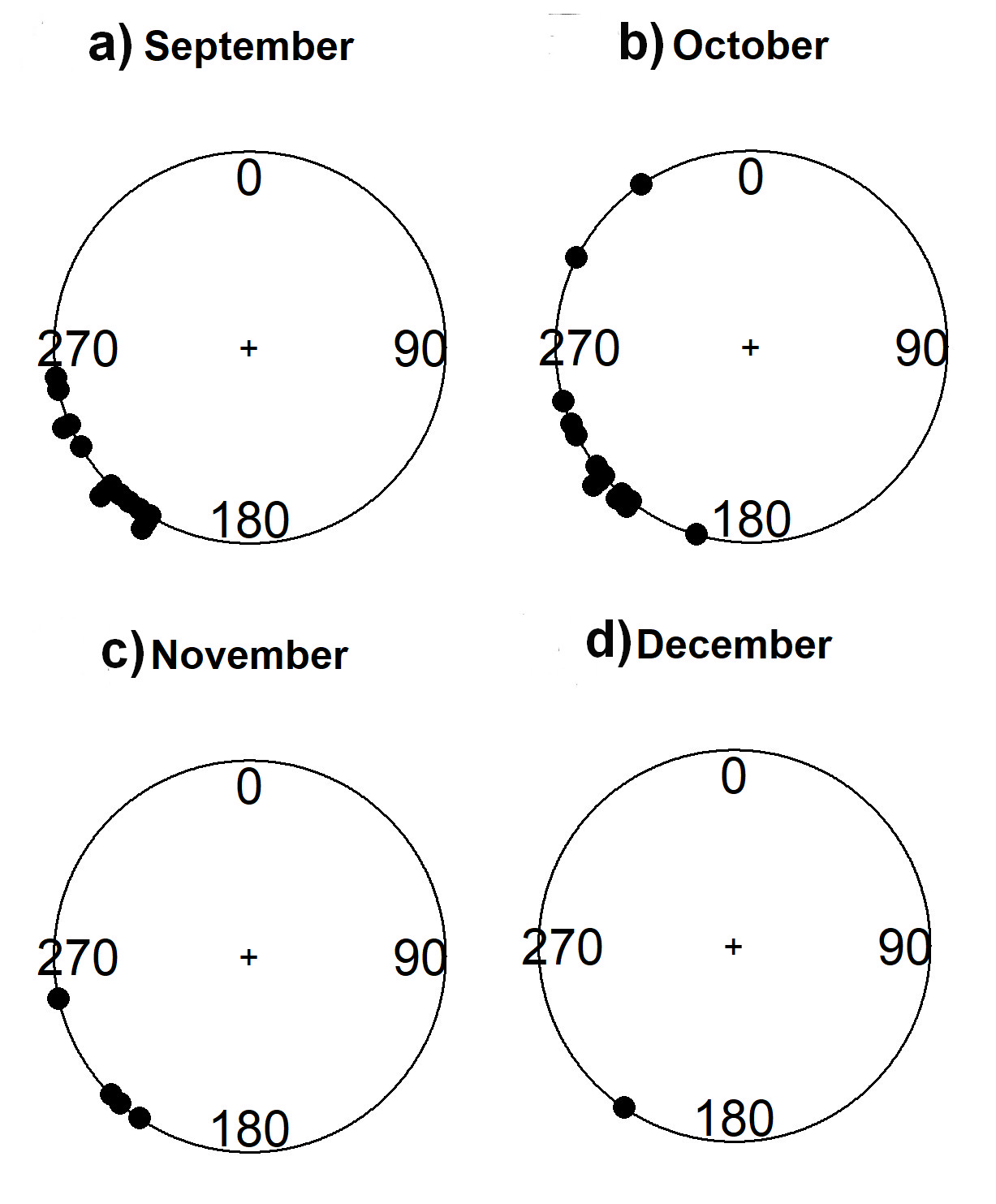

Supplement: Supplementary file 2 — Supplementary Figure 2. [file 41598_2020_73996_MOESM2_ESM.tif]

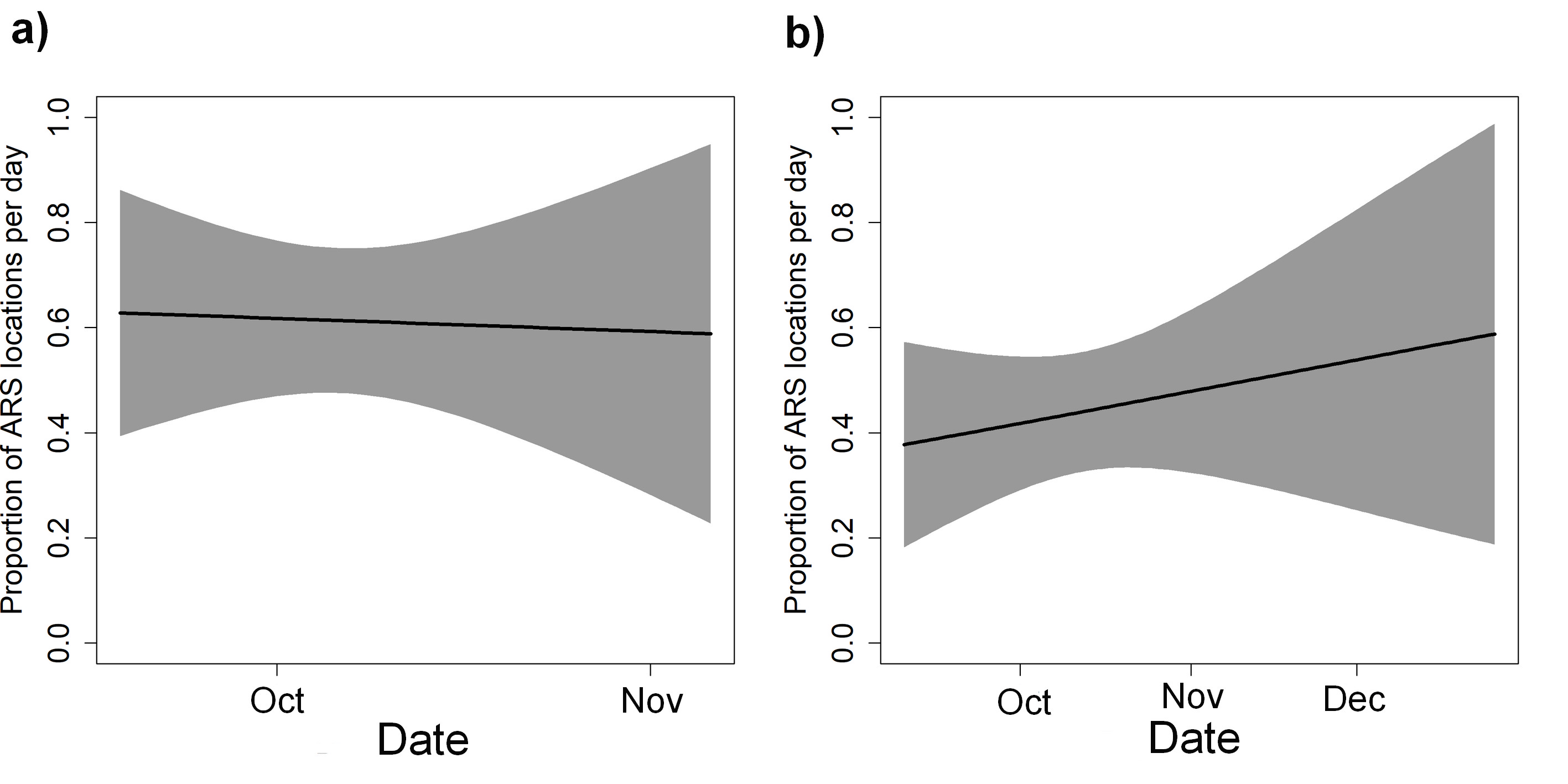

Supplement: Supplementary file 3 — Supplementary Figure 3. [file 41598_2020_73996_MOESM3_ESM.tif]

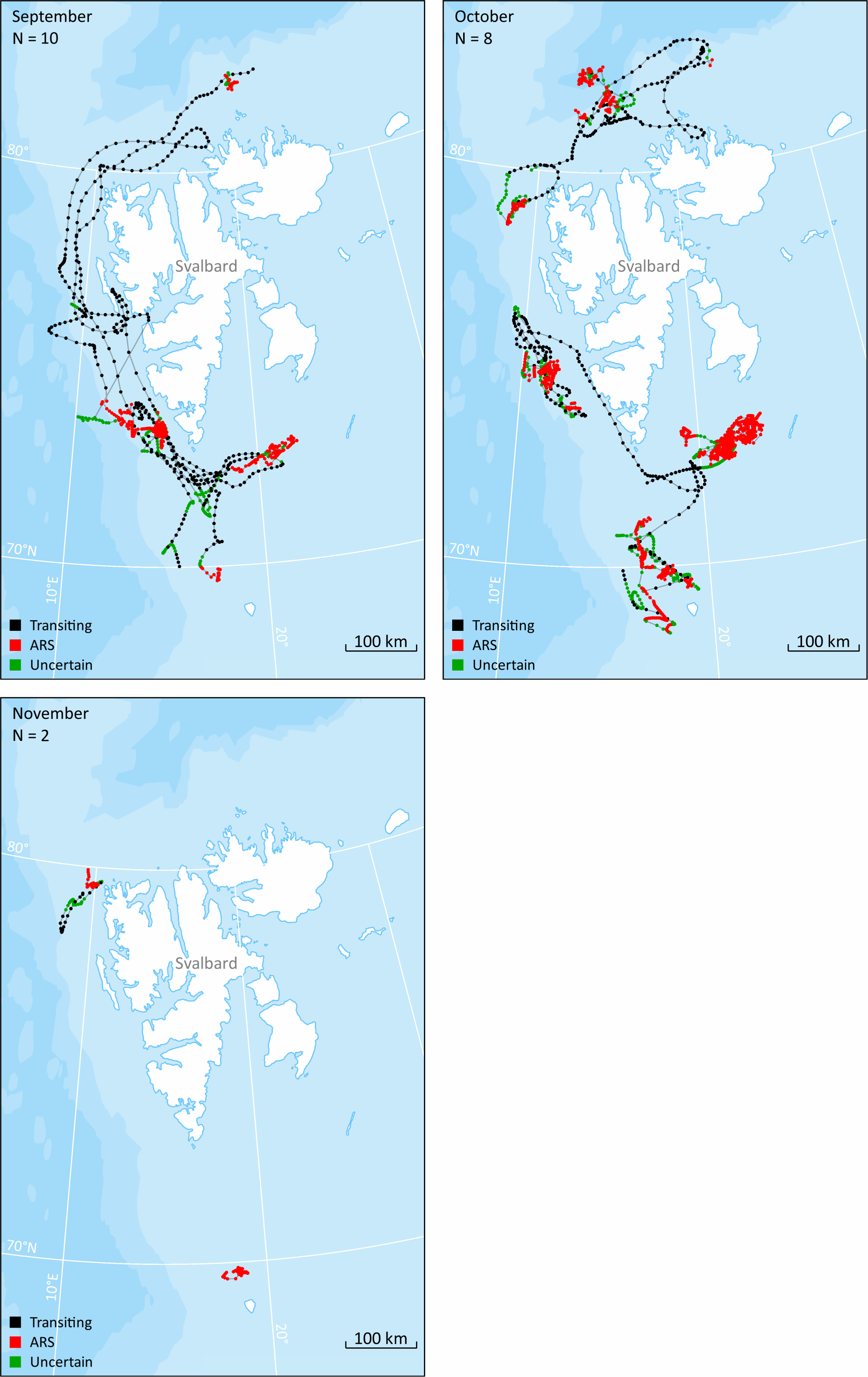

Supplement: Supplementary file 4 — Supplementary Figure 4. [file 41598_2020_73996_MOESM4_ESM.png]

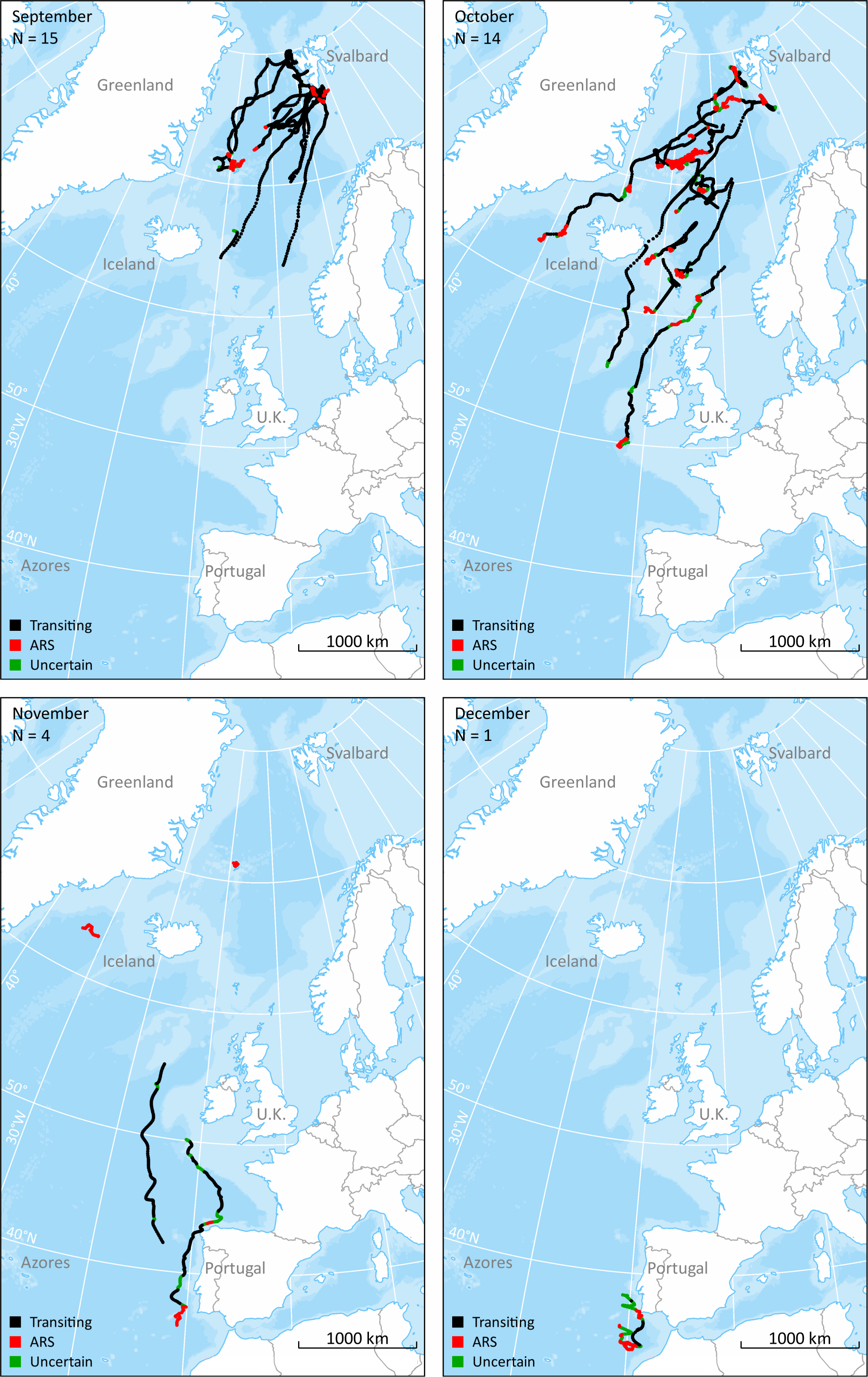

Supplement: Supplementary file 5 — Supplementary Figure 5. [file 41598_2020_73996_MOESM5_ESM.png]
