# Supplementary material for: Autumn movements of fin whales (Balaenoptera physalus) from Svalbard, Norway, revealed by satellite tracking
Source: Sci Rep. 2020 Oct 12;10:16966. doi: 10.1038/s41598-020-73996-z (PMC7550606; doi:10.1038/s41598-020-73996-z)
Supplement: Supplementary file 6 — Supplementary Legends. [file 41598_2020_73996_MOESM6_ESM.docx]

**Supplementary information.**

Title: Autumn movements of fin whales (*Balaenoptera physalus*) from Svalbard, Norway, revealed by satellite tracking.

Authors: Christian Lydersen, Jade Vacquié-Garcia, Mads Peter Heide-Jørgensen, Christophe Guinet, Nils Øien and Kit M. Kovacs

**Supplementary Figure 1.** Distribution of the movement parameter ɣ (i.e. movement persistence) used to infer behaviour (i.e. Area-restricted search (ARS) vs transiting) of fin whales tagged with satellite transmitters in the Svalbard area during September 2015, 2018 and 2019.

**Supplementary Figure 2**. Dispersion directions from departure locations for 15 fin whales that left the Svalbard area a) September, b) October, c) November and d) December**.**

**Supplementary Figure 3.** Seasonal change in the proportion of estimated locations considered as area-restricted search (ARS) for fin whales instrumented with satellite transmitters on the west coast of Svalbard during September 2015, 2018 and 2019. a) residency period and b) migratory period.

**Supplementary Figure 4.** Hierarchical switching state-space model derived locations and inferred behavioural modes on a monthly basis for 10 fin whales (instrumented with satellite tags on the west coast of Svalbard during September 2015, 2018 and 2019) that remained in Svalbard throughout their tracking periods. The maps were generated based on publicly available ArcMap polar projections documents using ArcGIS 10.1 ([www.esri.com](http://www.esri.com)).

**Supplementary Figure 5.** Hierarchical switching state-space model derived locations and inferred behavioural modes on a monthly basis for 15 fin whales (instrumented with satellite tags on the west coast of Svalbard during September 2015, 2018 and 2019) that undertook southward migrations during their tracking periods. The maps were generated based on publicly available ArcMap polar projections documents using ArcGIS 10.1 ([www.esri.com](http://www.esri.com)).
